# MicroRNA-155 Regulates MAIT1 and MAIT17 Cell Differentiation

**DOI:** 10.3389/fcell.2021.670531

**Published:** 2021-04-09

**Authors:** Tingting Liu, Jie Wang, Kalpana Subedi, Qijun Yi, Li Zhou, Qing-Sheng Mi

**Affiliations:** ^1^Department of Dermatology, Center for Cutaneous Biology and Immunology Research, Henry Ford Health System, Detroit, MI, United States; ^2^Shandong Provincial Hospital for Skin Diseases, Shandong Provincial Institute of Dermatology and Venereology, Shandong First Medical University, Shandong Academy of Medical Sciences, Jinan, China; ^3^Immunology Research Program, Henry Ford Cancer Institute, Henry Ford Health System, Detroit, MI, United States

**Keywords:** microRNA-155, mucosal-associated invariant T cells, MAIT1, MAIT17, differentiation

## Abstract

Mucosal-associated invariant T (MAIT) cells are innate-like T cells that develop in the thymus through three maturation stages to acquire effector function and differentiate into MAIT1 (T-bet^+^) and MAIT17 (RORγt^+^) subsets. Upon activation, MAIT cells release IFN-γ and IL-17, which modulate a broad spectrum of diseases. Recent studies indicate defective MAIT cell development in microRNA deficient mice, however, few individual miRNAs have been identified to regulate MAIT cells. MicroRNA-155 (miR-155) is a key regulator of numerous cellular processes that affect some immune cell development, but its role in MAIT cell development remains unclear. To address whether miR-155 is required for MAIT cell development, we performed gain-of-function and loss-of-function studies. We first generated a CD4Cre.miR-155 knock-in mouse model, in which miR-155 is over-expressed in the T cell lineage. We found that overexpression of miR-155 significantly reduced numbers and frequencies of MAIT cells in all immune organs and lungs and blocked thymic MAIT cell maturation through downregulating PLZF expression. Strikingly, upregulated miR-155 promoted MAIT1 differentiation and blocked MAIT17 differentiation, and timely inducible expression of miR-155 functionally inhibited peripheral MAIT cells secreting IL-17. miR-155 overexpression also increased CD4^–^CD8^+^ subset and decreased CD4^–^CD8^–^ subset of MAIT cells. We further analyzed MAIT cells in conventional miR-155 knockout mice and found that lack of miR-155 also promoted MAIT1 differentiation and blocked MAIT17 differentiation but without alteration of their overall frequency, maturation and function. Overall, our results indicate that adequate miR-155 expression is required for normal MAIT1 and MAIT17 cell development and function.

## Introduction

Mucosal-associated invariant T (MAIT) cells are innate-like T lymphocytes that are involved in antimicrobial immunity, acting as a bridge between the innate and adaptive immune responses. Recently, MAIT cells have captured the attention of immunologists and clinicians because they have been shown to be abundant in humans and affect a broad range of diseases, such as infectious disease, autoimmune disease, and cancer (Duan et al., 2019; Godfrey et al., 2019; Legoux et al., 2020; Rodin et al., 2020). MAIT cells express a semi-invariant T cell receptor (TCR) consisting of an invariant Vα chain (Vα19/Jα33 in mice and Vα7.2/Jα33 in humans) and a biased Vβ repertoire (Reantragoon et al., 2013; Lepore et al., 2014). These TCRs give MAIT cells the ability to recognize microbial riboflavin-derivative antigens presented by MHC class I-related (MR1) molecules (Kjer-Nielsen et al., 2012; Corbett et al., 2014; Legoux et al., 2019). MAIT cells are detectable in a broad range of tissues, and they are considerably more abundant in humans than in mice, especially in non-mucosal tissues, where they represent up to 10% of blood T cells and 45% of liver T cells (Dusseaux et al., 2011; Gherardin et al., 2018; Chen et al., 2019). In C57BL/6 mice, the highest frequency of MAIT cells in total αβT cells is detected in the skin (5–40%), followed by lungs (3.3%), lamina propria (0.7%), liver (0.6%), peripheral lymph nodes (0.2%), blood (0.09%), spleen (0.08%), and thymus (0.05%) (Rahimpour et al., 2015; Constantinides et al., 2019).

Mouse intra-thymic development of MAIT cells can be divided into three developmental stages based on expression of the surface markers CD24 and CD44 (Koay et al., 2016; Wang and Hogquist, 2016). Interaction between double-positive thymocytes expressing MR1 and those expressing TCRs generates stage 1 (CD24^+^CD44^–^) committed precursors; these cells are only found in the thymus and are the dominant subset in very young (2–4 weeks) mice. Then, these cells cease CD24 expression, migrate to the medulla, and develop into stage 2 (CD24^–^CD44^–^) cells. The final maturation step is the emergence of stage 3 (CD24^–^CD44^+^) cells that start to express classic markers (IL-18R and PLZF) and more closely resemble mature MAIT cells. Mature MAIT cells can be further divided into MAIT1 (T-bet^+^) and MAIT17 (RORγt^+^) sub-lineages. MAIT1 cells mainly secrete IFN-γ and preferentially localize to the liver and spleen, while MAIT17 cells produce IL-17 and are positioned in mucosal tissues, such as lung, skin, and gut lamina propria (Lantz and Legoux, 2019). Recently, Lee and colleagues proposed the existence of MAIT2 cells (CD24^–^PLZF^hi^), which mainly consist of stage 2 MAIT cells in the thymus, and these cells were considered as developmental intermediates of MAIT1 and MAIT17 cells (Lee et al., 2020). Based on the expression of CD4 and CD8, MAIT cells have four subsets, CD4^+^CD8^–^, CD4^–^CD8^+^, CD4^–^CD8^–^, and CD4^+^CD8^+^, and their frequency varies in a tissue- and strain-specific manner. Although the majority of MAIT cells are CD4^–^CD8^–^ in all tissues, a clear population of MAIT cells express either CD4 or CD8 can be observed in the thymus, spleen, lymph nodes, and liver in C57BL/6 mice (Rahimpour et al., 2015; Constantinides et al., 2019; Winter et al., 2019).

Upon stimulation, MAIT cells are rapidly activated and secrete cytokines such as IFN-γ and IL-17, mediating functions that link innate and acquired immunity in a broad spectrum of diseases, including infection, cancer, allergy, and autoimmunity (Godfrey et al., 2019). TCR-dependent and TCR-independent processes involved in the activation of MAIT cells exert overlapping and specific effector functions, and both activation pathways promote a broad production of pro-inflammatory cytokines (Keller et al., 2017; Godfrey et al., 2019; Hinks et al., 2019; Lamichhane et al., 2019; Leng et al., 2019; Hinks and Zhang, 2020). The cytokine and chemokine repertoire induced by MR1 ligand is dominated by IL-17A, whereas a more modest cytokine response dominated by IFN-γ is driven by IL-18 in synergy with IL-12 and IL-15 and is potentiated by TL1A [Tumor necrosis factor (TNF)-like protein 1A; Leng et al., 2019]. Moreover, TCR-triggered MAIT cells show specific enrichment of tissue repair functions (Hinks et al., 2019; Lamichhane et al., 2019; Leng et al., 2019; Salou and Lantz, 2019). In different species, strong similarities exist between human and mouse MAIT cells in terms of tissue repair signatures and pro-inflammatory cytokines and chemokines (Hinks et al., 2019; Leng et al., 2019).

Increasing studies have reported that several genes regulate the development and function of MAIT cells. Transcription factor (PLZF) (Rahimpour et al., 2015; Koay et al., 2016), cytokines (IL-1, IL-7, IL-12, IL-15, IL-18, IL-23) (Tang et al., 2013; Ussher et al., 2014; Sattler et al., 2015; Constantinides et al., 2019; Berkson et al., 2020), and other genes (MR1, CD1d, CCR7, CXCR6, ZAP70, SAP, CXCR6, SATB1, Traj33, Jα18, DGKα, DGKζ) (Koay et al., 2016, 2019a,b; Xie et al., 2019; Nejman et al., 2020) are involved in the development and activation of MAIT cells. Moreover, recent studies indicated that MAIT cell development is controlled by the microRNAs (miRNAs) (Koay et al., 2016; Koay and Godfrey, 2019; Winter and Krueger, 2019; Winter et al., 2019). miRNAs, a class of 21–25 nucleotide single-stranded non-coding small RNAs, are emerging as key regulators of numerous cellular processes that affect cell development, homeostasis, and disease development (Bartel, 2004). miRNAs repress their target genes post-transcriptionally, and one individual miRNA can potentially target multiple genes depending on the cellular context. After complete deletion of miRNAs in T cells through targeting *Drosha* (a member of the RNase III superfamily that initiates the processing of miRNA), most MAIT cells are arrested at stage 1 in the thymus, and failed to process to stage 2 and stage 3, suggesting that miRNAs control MAIT cell development beyond stage 1 (Koay et al., 2016). In miR-181a/b-1 (a pair of microRNAs that serve as a rheostat of TCR signal strength) deficient mice, frequency of MAIT cells is decreased in the thymus and peripheral organs; most MAIT cells are profoundly arrested at stage 1; functional maturation of MAIT cells is precluded; PLZF expression is reduced and differentiation of thymic MAIT cells is also affected (Winter et al., 2019).

miR-155 is highly expressed in monocytes/macrophages, B cells, and T cells, and upregulation of miR-155 has been shown to be a consistent feature of the mammalian inflammatory response (Vigorito et al., 2007; Teng et al., 2008). Abundant studies have reported the various roles of miR-155 in regulating the differentiation and response of multiple immune cells, including Th17 and regulatory T cells (Seddiki et al., 2014). Furthermore, we and others have reported that miR-155 plays an important role in invariant natural killer T (*i*NKT) cell development and function (Burocchi et al., 2015; Frias et al., 2018; Wang et al., 2021). However, the role of miR-155 in MAIT cell development and functional lineage differentiation remains unknown. To ascertain the potential role of miR-155 in MAIT cell development and function, two complementary approaches, gain-of-function and loss-of-function, were used. We first generated a CD4Cre.miR-155 knock-in (KI) mouse model, in which miR-155 is specifically over-expressed in the T cell lineage. We found that overexpression of miR-155 significantly reduced number and frequencies of MAIT cells in all lymphoid organs and lung, blocked thymic MAIT cell maturation, and increased CD8^+^ subset and decreased CD4^–^CD8^–^ subset of MAIT cells. Strikingly, upregulated miR-155 promoted MAIT1 differentiation and blocked MAIT17 differentiation, and inducible expression of miR-155 functionally inhibited peripheral MAIT cells secreting IL-17. We further analyzed MAIT cells in conventional miR-155 knockout mice and found that lack of miR-155 also promoted MAIT1 differentiation and blocked MAIT17 differentiation but without alteration of their frequency, maturation and function. Our results uncover miR-155 as a key epigenetic regulator for MAIT1 and MAIT17 cell development and function.

## Materials and Methods

### Mice

Conventional miR-155 KO in the C57BL/6J background and wild-type (WT) C57BL/6J mice were purchased from the Jackson Laboratory (Bar Harbor, ME). Mice carrying the miR-155KI.Rosa26.GFP allele (Jackson Laboratory) were bred with CD4Cre transgenic mice (Jackson Laboratory) to obtain mice expressing CD4Cre with one or two copies of the miR-155 KI allele. Mice with two copies of the miR-155 KI allele, miR-155KI mice (CD4Cre^+^miR-155KI, miR-155KI), and the WT littermate CD4Cre^–^miR-155 KI mice served as controls. Inducible miR-155 overexpression mice were generated by crossing miR155KI-GFP mice with ubiquitin C promoter-driven, tamoxifen-inducible Cre (UbcCreER) transgenic mice (Jackson Laboratory). Experiments were conducted on 8–15-week-old mice that matched for age and gender. Mice were housed in a specific pathogen-free barrier unit. Handling of mice and experimental procedures were in accordance with requirements of the Institutional Animal Care and Use Committee.

### Cell Preparation

Single-cell suspensions of thymus, spleen, and lymph nodes (LNs) were obtained by gently grinding each organ through a 40 μm filter. Red blood cells in spleen were lysed using red blood cell lysis buffer. For lymphocyte isolation from the lung and liver, the two organs were immediately perfused with phosphate-buffered saline (PBS) after mice were sacrificed. Lung tissues were cut into pieces and treated in digestion solution (PBS + 3%FBS + DNase [100 U/ml] + Collagenase D [1 mg/ml]) for 50 min at 37^°^C on a shaker. Hepatic leukocytes were isolated by density gradient centrifugation with 33% percoll medium (50 ml 33% percoll: 1.84 10 × PBS + 16.56 ml percoll + 31.6 ml RPMI medium) at 2,300 rpm at room temperature for 25 min without acceleration or brake.

### Flow Cytometry

Single-cell suspensions were washed with staining buffer (1X PBS, 2% FBS) and incubated with Fc block (clone 2.4G2). Cells were stained with MR1-tetramers (provided by the NIH Tetramer Core Facility). The following conjugated mAbs were used: TCRβ (H57–597), CD24 (30-F1), CD44 (IM7), PLZF (9E12), RORγt (B2D), T-bet (eBio4B10), CD8 (53-6.7), CD4 (RM4-5), IFN-γ (XMG1.2), and IL-17a (eBio17B7). All mAbs were purchased from eBioscience, Biolegend, or TONBO. Cell surface staining was performed with staining buffer. Intranuclear staining for PLZF, T-bet, and RORγt was performed with eBioscience Fixation/permeabilization buffer. The flow cytometry assay was performed through BD FACSCelesta and data were analyzed using FlowJo V10.2 software. The gating strategy was as follows: after gating on lymphocyte, doublets were excluded by using forward scatter (FSC) and side scatter (SSC), and MAIT cells were identified as TCRβ^+^ MR1-tetramer^+^.

### Tamoxifen Treatment

Tamoxifen (Sigma Aldrich, St. Louis, MO) was dissolved in corn oil (Sigma Aldrich) containing 10% (vol/vol) ethanol (Thermo Fisher Scientific) and was intraperitoneally administered at 50 mg/kg of mouse weight for five consecutive days.

### *In vitro* Functional Assays

Single-cell suspensions from spleen, LNs, liver and lung were cultured in T cell culture medium (RPMI 1640 with 10% FBS, HEPES, penicillin and streptomycin, pyruvate, non-essential amino acids, L-glutamine, and 2-ME) in the presence of phorbol 12-myristate 13-acetate (PMA) (50 ng/ml) and Ionomycin (1 μM) for a total of 4 h at 37 ^°^C. Brefeldin A was then added in the last 2.5 h at a final concentration of 1 μM. Anti-IFN-γ and anti-IL-17 were detected by intracellular staining and flow cytometry.

### MAIT Cell Enrichment

Total thymocytes were stained with APC-conjugated MR1 tetramer, bound to anti-APC MicroBeads (Miltenyi Biotec), and enriched on an autoMACS Pro Separator (Miltenyi Biotec) using the POSSEL_S program (Constantinides et al., 2019). Positively selected cells were then stained with surface and intracellular antibodies to examine the developmental stage and functional linage.

### Real-Time RT-PCR

RNA from thymic CD4^+^ T cells from miR-155 KI and WT counterpart mice was purified by using the EXIQON isolation. RNA was reverse transcribed using the PrimeScriptTM RT reagent kit, and RT-qPCR was performed on an Applied Biosystems 7900 Real-Time PCR. Expression levels of miR-155 were normalized with snoRNU202, and fold change was calculated using the 2^–ΔΔCT^ method.

### Statistical Significance

GraphPad Prism 8.0 was used for statistical analysis (unpaired, two-tailed, *t*-test with a confidence interval of 95%). A *p* < 0.05 was considered statistically significant.

## Results

### Reduced Frequency and Number of MAIT Cells in CD4Cre miR-155 KI Mice

**FIGURE 1:**
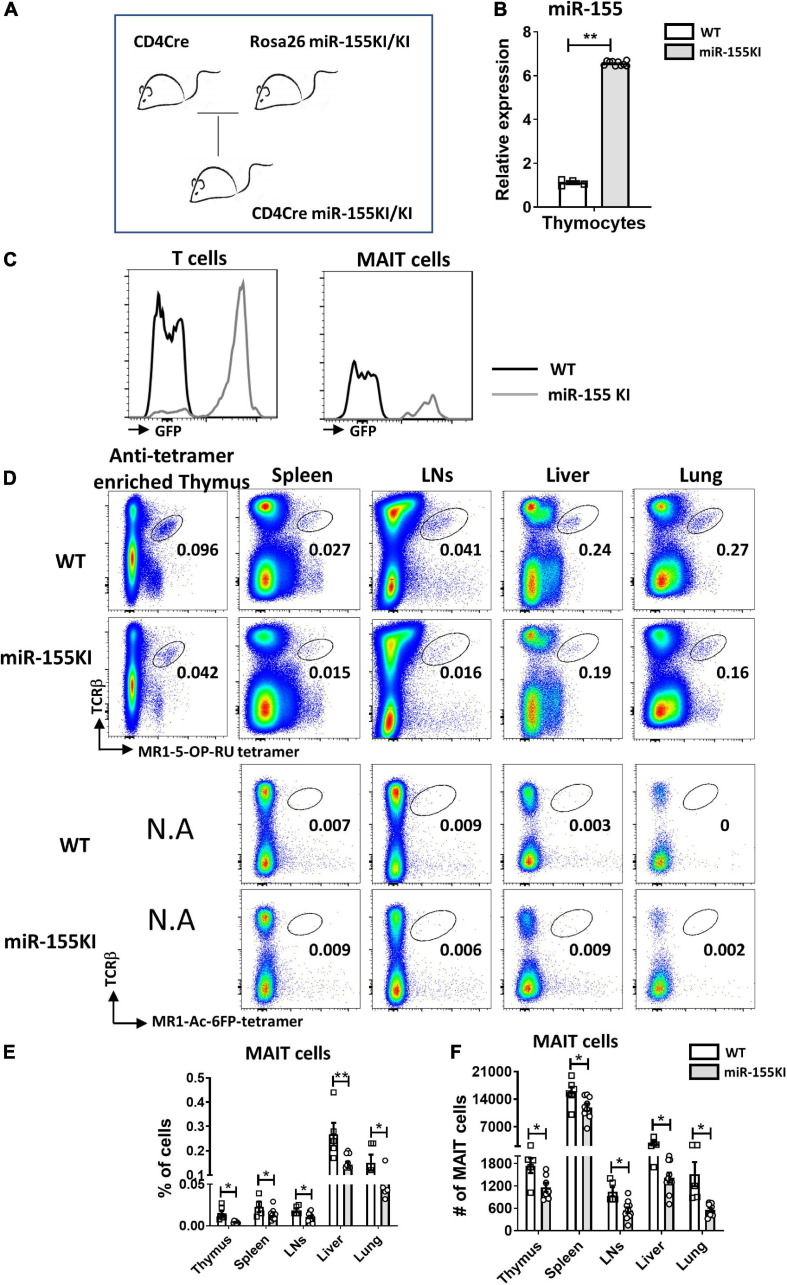
miR-155 overexpression interferes MAIT cell development. **(A)** CD4Cre miR-155 KI mouse model. **(B)** Thymic T cells were harvested from WT controls and miR-155 KI mice. Total RNA was purified and miR-155 and snoRNU202 expression was measured by RT-PCR. **(C)** Histogram showing GFP expression of T cells and MAIT cells in miR-155KI mice. **(D)** Defective MAIT cell development in miR-155 KI mice. Representative flow cytometric plots showing the frequencies of MAIT cells in the thymus, spleen, LNs, liver, and lung of WT and miR-155 KI mice. Numbers adjacent to outlined areas indicate frequency of indicated populations. **(E)** The percentage and **(F)** the absolute numbers of MAIT cells in indicated organs from miR-155 KI mice. Data are from at least three independent experiments. Each mouse is represented as one dot. 3–8 mice for each group. Data were analyzed by unpaired *t*-test. *p < 0.05, **p < 0.01 compared to WT.

To dissect the role of miR-155 on MAIT cell development, a gain-of-function study was first performed using mice with T cell-specific overexpression of miR-155. Mice carrying the miR-155KI.Rosa26.GFP KI allele were bred with CD4Cre transgenic mice to generate mice overexpressing the miR-155KI allele in T cell linage (CD4Cre^+^miR-155KI, miR-155KI; Figure 1A). Substantial upregulation of miR-155 was confirmed by RT-PCR in sorted TCRβ^+^ T cells from thymus in miR-155 KI mice (Figure 1B). Given that the T cells from miR-155 KI mice were GFP positive, we therefore used flow cytometry to further confirm the overexpression of miR-155. As expected, we found that miR-155-GFP was indeed upregulated in T cells and MAIT cells (Figure 1C).

The frequency and number of MAIT cells were measured in the different immune and non-immune organs from miR-155 KI and WT mice, including thymus, spleen, LNs, liver, and lungs by flow cytometry, based on the surface staining of TCRβ and 5-OP-RU-MR1 tetramers. Due to the rareness of MAIT cells in the thymus, we enriched MAIT cells from total thymocytes using APC-conjugated MR1-tetramer. We found significant reduction in the frequencies of thymic and peripheral MAIT cells in miR-155 KI mice, with an approximately twofold reduction in all organs (Figures 1D,E). As expected, a significant decrease in the absolute number of MAIT cells was also detected in these organs (Figure 1F). Thus, overexpression miR-155 impairs MAIT cell development.

### Overexpression of miR-155 Blocks Thymic MAIT Cell Maturation

**FIGURE 2:**
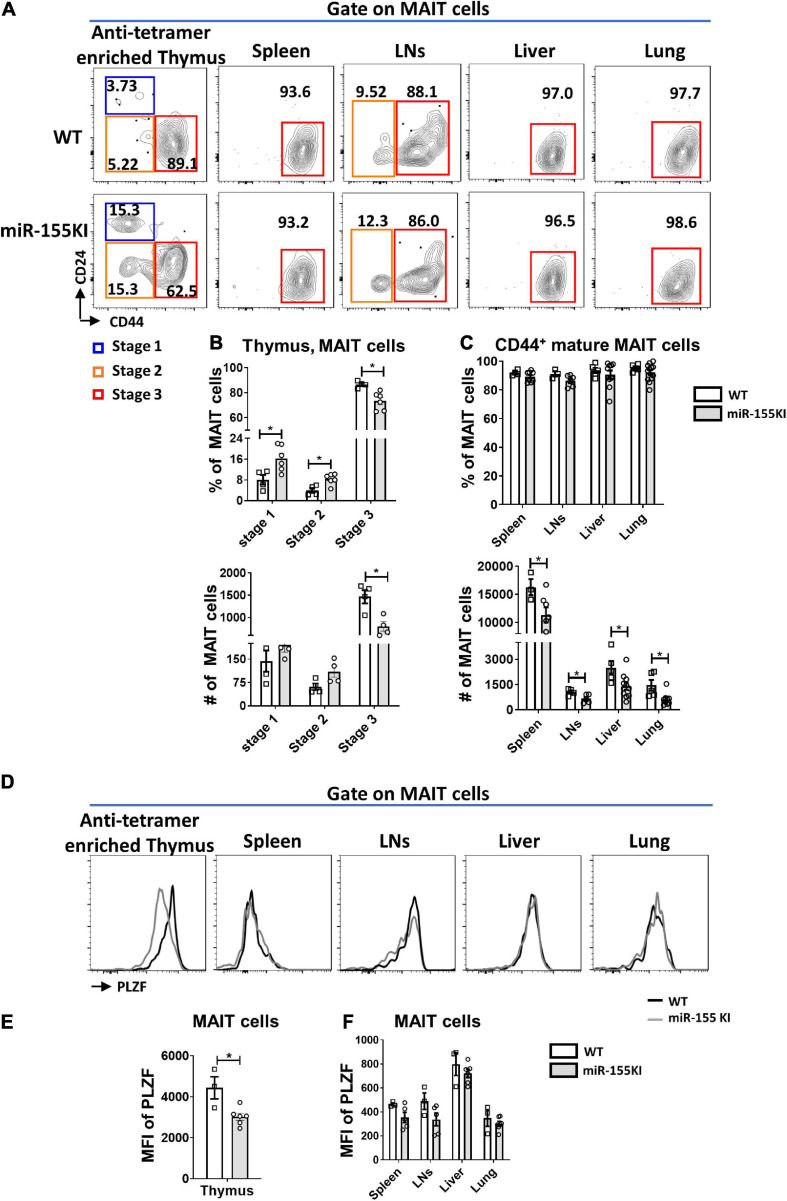
Role of miR-155 in MAIT cell maturation. **(A)** The developmental stages of thymic and peripheral MAIT cells were assessed by staining the surface expression of CD24 and CD44 gated on MAIT cells. **(B)** Frequency and absolute number of enriched thymic MAIT cells at each stage of development and **(C)** frequency and absolute number of CD44^+^ mature MAIT cells in peripheral organs. **(D)** Histograms showing PLZF expression by gated MAIT cells in indicated organs of WT (black outline) and miR-155 KI (gray) mice. **(E)** Expression of PLZF in thymic and **(F)** peripheral MAIT cells. Data are from two independent experiments. Each mouse is represented as one dot. 3–8 mice for each group. Data were analyzed by unpaired *t*-test. *p < 0.05 compared to WT.

To precisely explore the effect of miR-155 on MAIT cell maturation, cell surface markers distinguishing the MAIT cell maturation stages were assessed in thymic and peripheral MAIT cells. The frequencies of stage 1 (CD24^+^CD44^–^) and stage 2 (CD24^–^CD44^–^) MAIT cells were significantly increased in the thymus, however, the frequency of stage 3 (CD24^–^CD44^+^) MAIT cells were significantly reduced in miR-155 KI mice compared to WT mice. However, the absolute numbers of stages 1, and 2 cells in miR-155KI mice were comparable with those in WT mice, whereas stage 3 MAIT cells were dramatically reduced in miR-155KI mice (Figures 2A,B). Interestingly, unlike MAIT cells in thymus, the frequencies of mature MAIT cells were comparable in spleen, LNs, liver and lung between miR-155 KI mice and WT mice, but the absolute numbers were decreased dramatically (Figure 2C).

A previous study has reported that ~70% of thymic MAIT cells express the transcription factor PLZF, similar to the frequency of mature stage 3 MAIT cells (Winter et al., 2019). Given that miR-155 affected thymic MAIT cell maturation, we therefore test PLZF expression. Consistently, the expression of PLZF in thymic MAIT cells was decreased dramatically in miR-155 KI mice compared to WT mice (Figures 2D,E), suggesting that miR-155 may control thymic MAIT cell development at least through targeting PLZF. However, the up-regulation of miR-155 did not significantly alter PLZF expression in MAIT cells from peripheral organs, which is consistent with the unaltered maturation of peripheral MAIT cells in miR-155 KI mice compared to WT mice (Figures 2D,F).

### Overexpression of miR-155 Promotes MAIT1 and Blocks MAIT17 Differentiation

**FIGURE 3:**
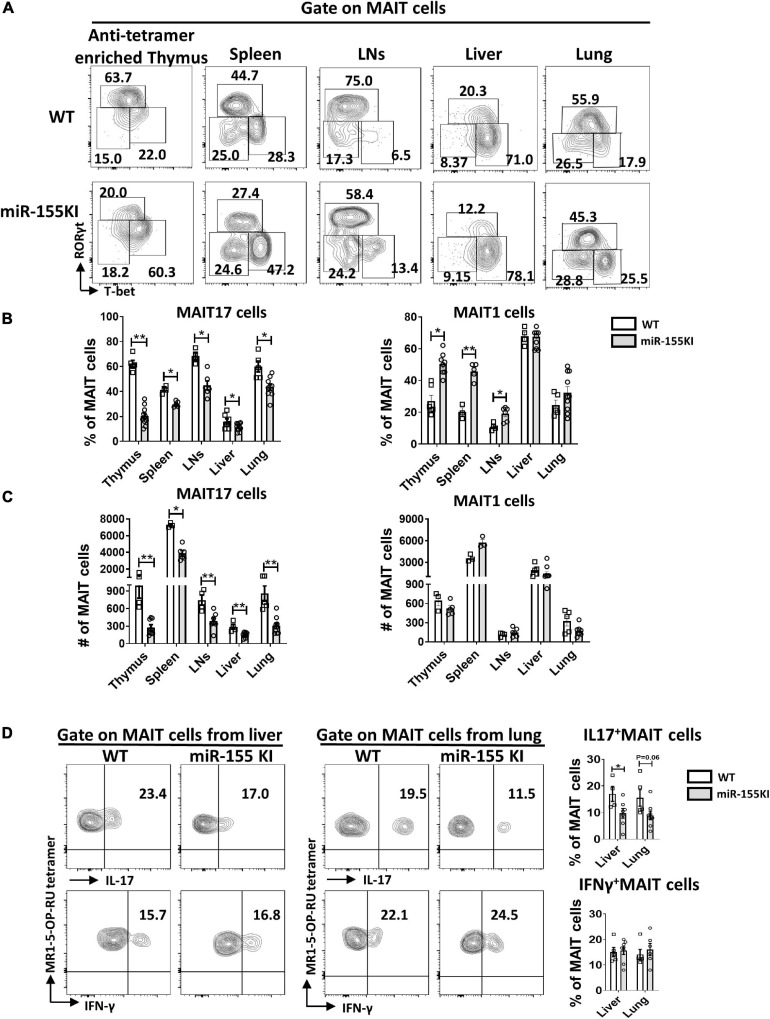
miR-155 overexpression regulates MAIT 1 and MAIT17 differentiation. **(A)** Expression of transcription factors in thymus and peripheral organs of MAIT cells from WT and miR-155 KI mice examined by intracellular staining of RORγt and T-bet. MAIT17 cells are defined as RORγt^+^ T-bet^–^ subsets and MAIT1 cells are defined as RORγt^–^ T-bet^+^ subsets. Numbers adjacent to outlined areas indicate percentage of indicated populations. **(B)** The percentage and **(C)** absolute number of MAIT17 and MAIT1 cells in thymus and peripheral organs. **(D)** IL-17 and IFN-γ production of MAIT cells from liver and lung in miR-155 KI mice. Data are from at least two independent experiments. Each mouse is represented as one dot. 3–8 mice for each group. Data were analyzed by unpaired *t*-test. *p < 0.05, **p < 0.01 compared to WT.

Thymic MAIT cells at stage 3 are functionally mature cells that have the potential to secrete cytokines and express the effector-associated transcription factors PLZF, RORγt, and T-bet. Recent studies showed that MAIT1 (T-bet^+^) cells preferentially localize in the liver, while MAIT17 (RORγt^+^) cells are positioned in mucosal tissues such as lung, skin, and gut lamina propria (Lantz and Legoux, 2019; Salou et al., 2019). To evaluate the potential role of overexpressing miR-155 in MAIT cell functional lineage differentiation, MAIT cells from the thymus, spleen, LNs, liver, and lung from miR-155 KI mice and WT littermates were analyzed for MAIT1 and MAIT17 differentiation. We found that the frequency of thymic MAIT17 cells was significantly reduced and the frequency of MAIT1 cells was increased dramatically in miR-155 KI mice (Figures 3A,B). Consistent with the thymic phenotype, miR-155 KI mice exhibited a remarkably decreased frequency of MAIT17 cells in peripheral organs, including spleen, LNs, liver, and lung (Figures 3A,B). The frequency of peripheral MAIT1 cells in miR-155 KI mice was significantly increased in the spleen and LNs compared to WT mice (Figure 3B). The absolute number of MAIT17 decreased dramatically, but the number of MAIT1 cells was comparable in miR-155 KI mice (Figure 3C). To characterize the function of MAIT cells, the cells of liver and lungs from miR-155KI and WT mice were stimulated with PMA/Ionomycin for 4 h *in vitro*. IL-17 producing MAIT cells were significantly decreased in the liver but not in the lung. We did not observe any significant changes in IFN-γ-producing MAIT cells between KI and WT mice, which was consistent with their differentiation phenotype (Figure 3D). Thus, MAIT cells with miR-155 overexpression showed interrupted MAIT cell differentiation, indicating an essential role for miR-155 in MAIT1 and MAIT17 cell functional lineage differentiation.

### miR-155 Overexpression Increases CD8^+^ MAIT Subset but Decreases CD4^–^CD8^–^ MAIT Subset

**FIGURE 4:**
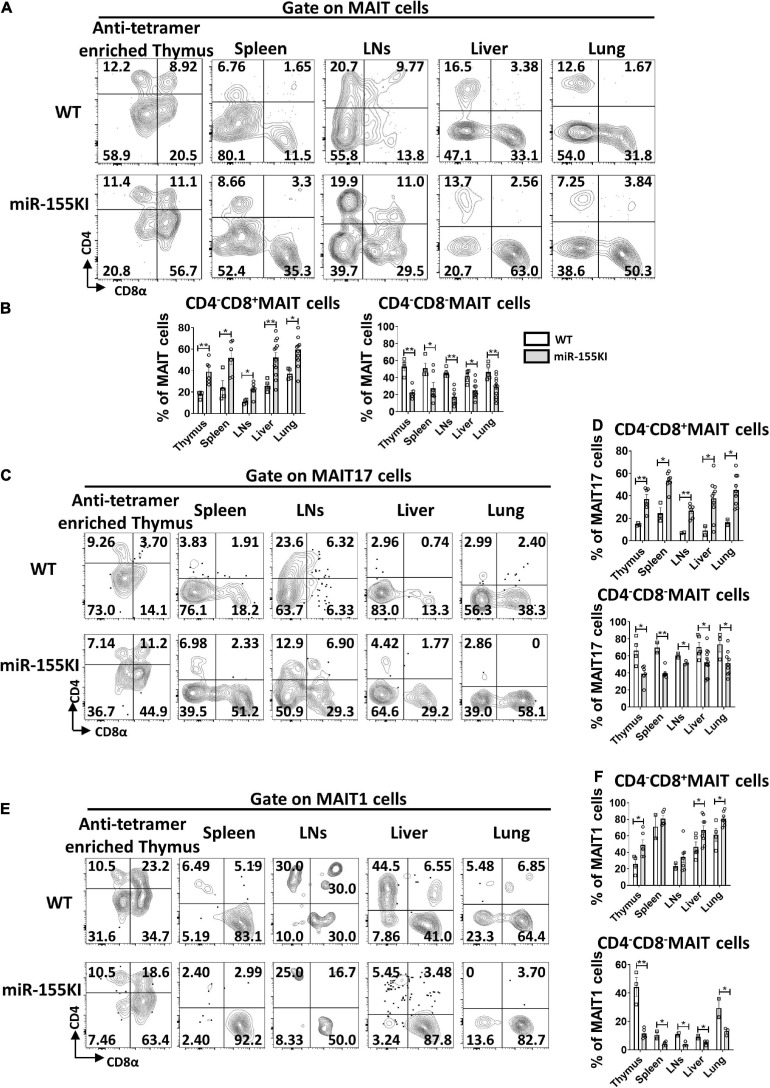
miR-155 increases CD4^–^CD8^+^ subset and decreases CD4^–^CD8^–^ subset in thymic and peripheral MAIT cells. **(A)** Flow cytometry analysis of CD4 and CD8 coreceptor expression on MAIT cells from thymus, spleen, LNs, liver, and lung from WT and miR-155 KI mice. **(B)** Percentage of MAIT cell subsets that express CD8, or neither coreceptor [CD4^–^CD8^–^] in the indicated organs. **(C)** Analysis of CD4 and CD8 expression on MAIT17 cells. **(D)** Percentages of CD4^–^CD8^+^ subset and CD4^–^CD8^–^ subset in MAIT17 cells. **(E)** CD4 and CD8 coreceptor expression pattern on MAIT1 cells in the indicated organs. **(F)** Percentages of CD4^–^CD8^+^ subset and CD4^–^CD8^–^ subset in MAIT1 cells. Data are from at least three independent experiments. Each mouse is represented as one dot. 3–8 mice for each group. Data were analyzed by unpaired *t*-test. *p < 0.05, **p < 0.01 compared to WT.

Although antigen recognition by MAIT cells does not rely on CD4 or CD8 coreceptors, MAIT cells do show heterogeneous expression of CD4 and CD8, with 4 different MAIT cell subsets, CD4^+^CD8^–^, CD4^–^CD8^+^, CD4^–^CD8^–^, and CD4^+^CD8^+^ in the thymus, while 3 major subsets CD4^+^CD8^–^, CD4^–^CD8^+^, CD4^–^CD8^–^ MAIT cells in peripheral tissues. In thymus, CD4 is highly expressed on stage 1 and 2 MAIT cells but is rarely expressed on most mature stage 3 MAIT cells. CD4^+^ stage 3 MAIT cells are likely to represent newly arrived stage 3 cells that have not yet committed to either MAIT1 or MAIT17 cells with functional maturation (Koay et al., 2019b). CD4^–^CD8^–^ MAIT cell subset is almost exclusively in barrier sites, including skin, lung, and gut, suggesting that double negative MAIT cells at barrier tissues may represent the most mature developmental stage (Rahimpour et al., 2015; Constantinides et al., 2019; Winter et al., 2019). Analysis of coreceptor expression on MAIT cells in miR-155 KI and WT mice revealed that CD4^+^CD8^–^ and CD4^+^CD8^+^ subsets were almost equivalent (data not shown), but increased frequencies of CD4^–^CD8^+^ cells and reduced frequencies of CD4^–^CD8^–^ cells were observed in miR-155 over-expressing MAIT cells from thymus, spleen, LNs, liver, and lung (Figures 4A,B).

To ascertain whether the impairment of MAIT cell sub-lineage differentiation in miR-155 KI mice is associated with CD4 and CD8 coreceptor expression, we examined the expression of CD4 and CD8 in MAIT17 and MAIT1 cells. As shown in Figures 4C–F, the CD4^–^CD8^–^ cell subset was abundant in MAIT17 cells and the CD4^–^CD8^+^ cell subset was enriched in MAIT1 cells in the thymus, spleen, LNs, liver, and lung; whereas the decreased CD4^–^CD8^–^ subset and increased CD4^–^CD8^+^ subset were observed both in miR-155 over-expressing MAIT1 and MAIT17 cells from thymus and peripheral organs (Figures 4C–F). These data implied that the alteration of CD4 and CD8 coreceptors may be involved in the interruption of MAIT cell sub-lineage differentiation in miR-155 KI mice.

### Overexpression of miR-155 Inhibits MAIT Cell Secreting IL-17

**FIGURE 5:**
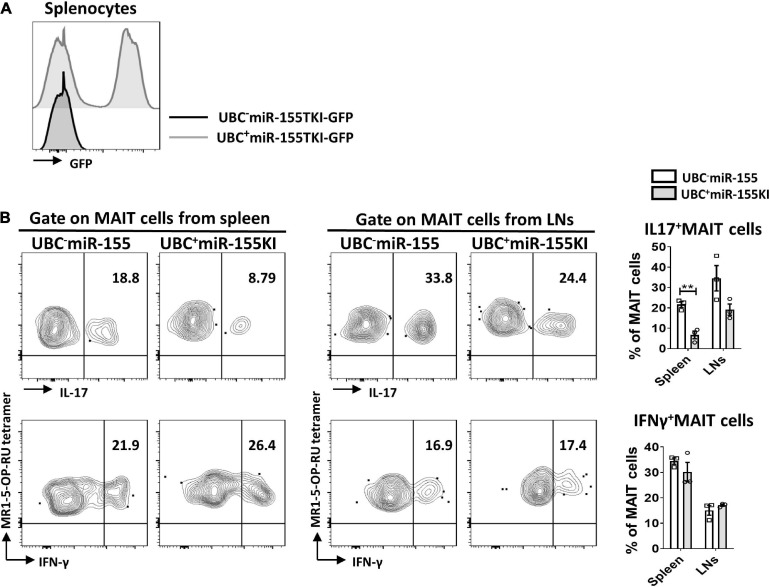
miR-155 is essential to the function of MAIT cells. **(A)** Histogram showing GFP expression of splenocytes in UBC^–^ miR-155 KI mice and UBC^+^ miR-155 KI mice after tamoxifen treatment. **(B)** Cytokines (IL-17 and IFN-γ) production of MAIT cells from spleen and LNs in UBC miR-155 KI mice. Data are from two independent experiments. Each mouse is represented as one dot. 3 mice for each group. Data were analyzed by unpaired *t*-test. **p < 0.01 compared to WT.

In order to bypass early developmental defects and delineate the impact of miR-155 in MAIT cell function, we generated inducible UBC^+^miR155KI-GFP mice by breeding miR155KI-GFP mice with a mouse expressing tamoxifen-inducible Cre (UBC^–^Cre ER mice). The UBC^–^ miR155KI-GFP mice and UBC^+^ miR155KI-GFP mice were treated with tamoxifen for 5 consecutive days, flow cytometry was used to confirm the up-regulation of miR-155 in splenocytes by GFP expression 9 days after tamoxifen treatment (Figure 5A). Following PMA/Ionomycin stimulation, we analyzed IL-17 and IFN-γ secreting MAIT cells, and we found significant reduction of IL-17-secreting MAIT cells from spleen, but IFN-γ-secreting MAIT cells were comparable both in spleen and LNs between UBC^–^ miR155KI-GFP mice and UBC^+^ miR155KI-GFP mice (Figure 5B), indicating that timely inducible expression of miR-155 inhibits function of peripheral MAIT cells.

### miR-155 Deficiency Does Not Affect MAIT Cell Development but Interrupts Thymic MAIT1 and MAIT17 Cell Differentiation

**FIGURE 6:**
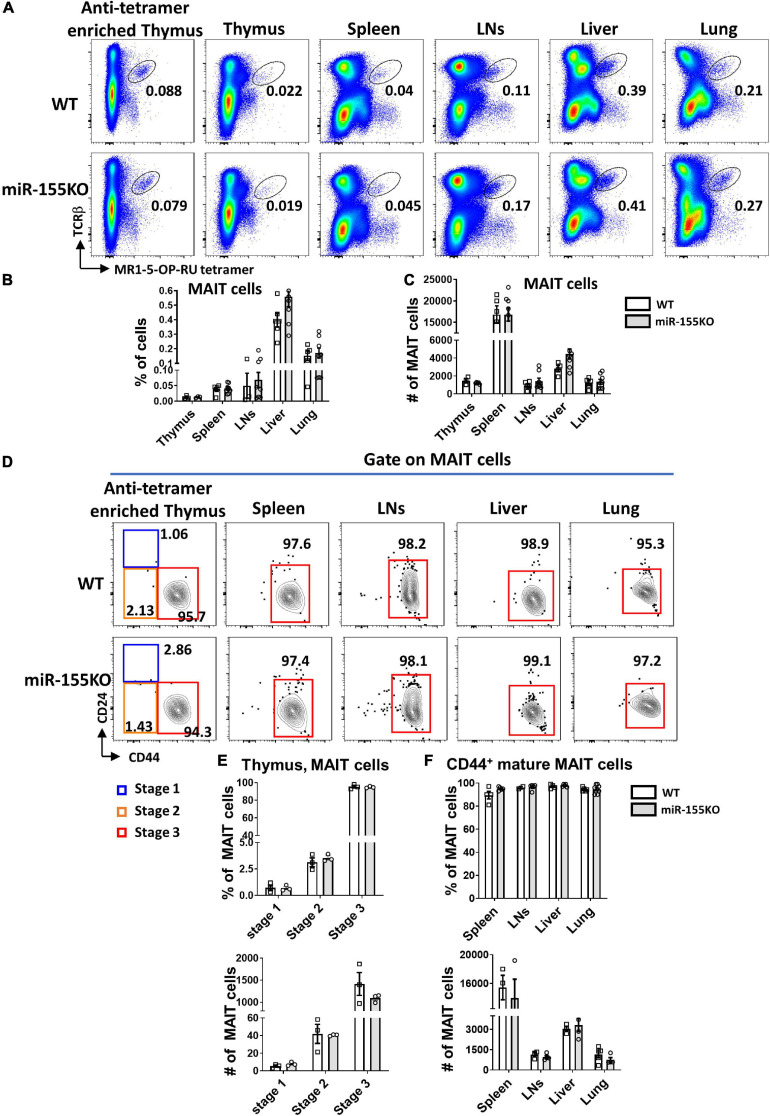
miR-155 deletion results in unchanged MAIT cell development. **(A)** Flow cytometric plots showing the frequencies of MAIT cells in the thymus, spleen, LNs, liver, and lung of WT and miR-155 KO mice. Numbers indicate percentage of cells within the drawn gate. Graph shows **(B)** the percentage and **(C)** absolute numbers of MAIT cells in the indicated organs of miR-155 KO mice. **(D)** Flow cytometry plot indicating CD24 and CD44 expression gated on MAIT cells from thymus and peripheral organs. **(E)** Percentage and absolute number of stage 1, stage 2 and stage 3 MAIT cells in enriched thymus. **(F)** Frequency and absolute number of CD44^+^ mature MAIT cells in peripheral lymphocytes. Data are from at least three independent experiments. Each mouse is represented as one dot. 3 mice for each group. Data were analyzed by unpaired *t*-test.

To further dissect the role of miR-155 on MAIT cell development, a loss-of-function study was performed using conventional miR-155 knockout (KO) mice and age- and sex-matched WT mice. The frequencies and absolute numbers of MAIT cells in thymus, spleen, LNs, liver, and lung peripheral organs were comparable between miR-155 KO and WT mice (Figures 6A–C). The frequencies and absolute numbers of MAIT cells at different mature stages in miR-155 KO were unaltered compared to those in WT mice based on CD24 and CD44 expression (Figures 6D–F).

**FIGURE 7:**
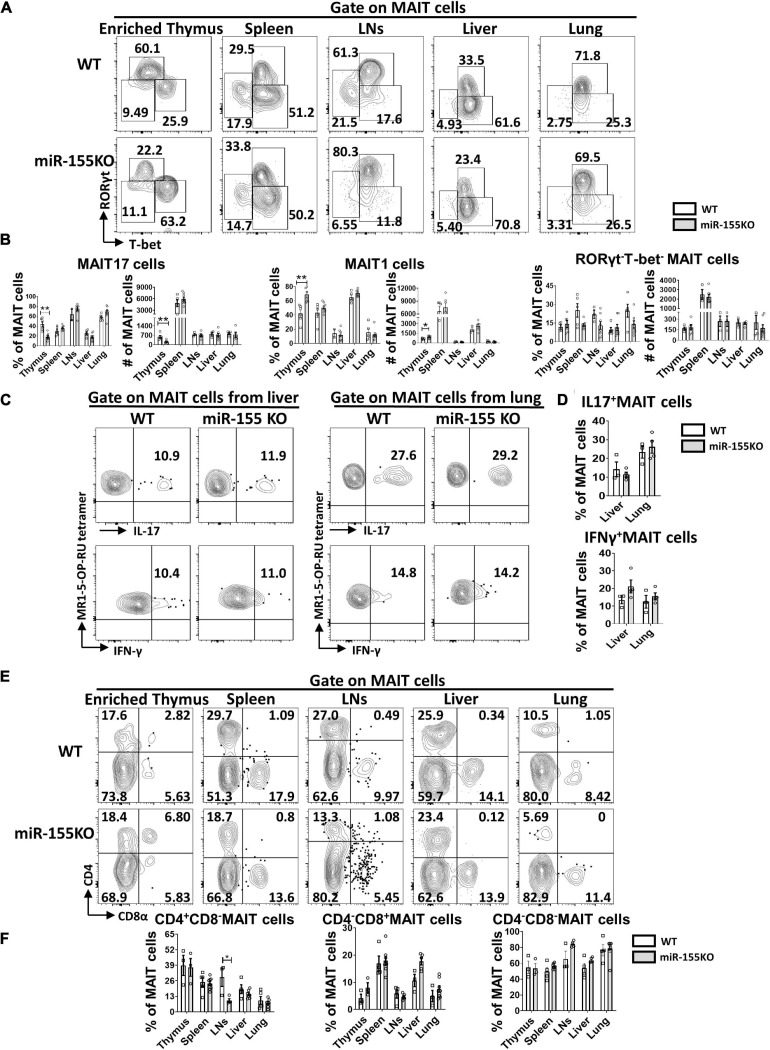
Differentiation and function of MAIT cells were unaffected substantially by the absence of miR-155. **(A)** Flow cytometric analysis of intracellular RORγt and T-bet expression in MAIT cells from thymus, spleen, LNs, liver, and lung in WT and miR-155KO mice. Numbers adjacent to outlined areas indicate percentage of indicated populations. **(B)** The percentages and absolute numbers of MAIT 17, MAIT1, RORγt^–^Tbet^–^ subsets in thymus and peripheral organs. **(C)** IL-17 and IFN-γ production of MAIT cells from liver and lung in miR-155 KO mice. **(D)** Percentage of IL-17 and IFN-γ in liver and lung. **(E)** Flow cytometry analysis of CD4 and CD8 coreceptor expression on MAIT cells in thymus, spleen, LNs, liver, and lung from WT and miR-155KO mice. **(F)** Percentage of MAIT cells subsets that express CD4, CD8, or neither coreceptor in the indicated organs. Data are from at least three independent experiments. Each mouse is represented as one dot. 3–8 mice for each group. Data were analyzed by unpaired *t*-test. *p < 0.05, **p < 0.01 compared with WT controls.

Next, the effect of miR-155 deletion on functional lineage differentiation was assessed both in thymus and peripheral organs. In the thymus, MAIT17 subset was dramatically decreased and MAIT1 was increased remarkably in miR-155 deficiency mice. However, no dramatic changes of MAIT1 and MAIT17 were observed in MAIT cells from peripheral organs in miR-155 KO mice. For the RORγt^–^Tbet^–^ subsets, no significant difference was found both in thymus and periphery (Figures 7A,B). Following PMA/Ionomycin stimulation, IL-17 and IFN-γ secretion were comparable in MAIT cells from liver and lung between miR-155KO and WT mice (Figures 7C,D). The frequencies of CD4^+^CD8^–^, CD4^–^CD8^+^, and CD4^–^CD8^–^ subsets in most organs were equivalent between miR-155KO and WT mice, but a significant decrease of CD4^+^CD8^–^ cells was detected in LNs from miR-155KO mice (Figures 7E,F). Taken together, deficiency of miR-155 promoted MAIT1 differentiation and blocked MAIT17 differentiation but without alteration of their overall frequency, maturation and function.

## Discussion

MAIT cells are a special lineage of T lymphocytes that go through a distinct developmental pathway controlled by a unique gene expression pattern (Koay et al., 2016). Due to the difficulties in identifying them in mice, the biology and function of mouse MAIT cells remain poorly defined, even though they have been recognized for more than 20 years (Porcelli et al., 1993; Tilloy et al., 1999). In the current study, we demonstrated a pivotal role for miR-155 in the development and differentiation of mouse MAIT cells by gain-of-function and loss-of-function studies. We found that overexpression of miR-155 impaired MAIT cell development, blocked MAIT cell maturation, interrupted MAIT1 and MAIT17 differentiation as well as impaired IL-17 secretion upon activation. Whereas lack of miR-155 did not dramatically affect MAIT cell development and function, but only impaired thymic MAIT1 and MAIT17 differentiation.

miRNAs are non-coding RNA molecules that target hundreds of mRNAs and influence the expression of many genes by epigenetic regulation. In mice with a deletion of the miRNA processing enzyme *Drosha*, thymic MAIT cells are primarily arrested at stage 1, while stage 3 MAIT cells are diminished in spleen and lymph nodes, suggesting that a complete loss of miRNAs affects maturation of MAIT cells (Koay et al., 2016). Deficiency of miR-181a/b impairs the development and functional maturation of MAIT cells (Winter et al., 2019). miR-155 is highly expressed in many hematopoietic cell types, including B and T cells and monocytes/macrophages (Landgraf et al., 2007), and has been identified as having multiple roles in regulating the differentiation and responses of various immune cells. Absence of miR-155 resulted in diminished germinal center responses and impaired B cell memory formation (Thai et al., 2007; Vigorito et al., 2007), failed induction of efficient CD4^+^ and CD8^+^ T cell activation (Rodriguez et al., 2007; Tsai et al., 2013), and reduced numbers and diminished proliferative potential of regulatory T cells (Lu et al., 2009). Additionally, recent studies have suggested that miR-155 also plays a role in *i*NKT cell development and function. miR-155 overexpression blocked the maturation of thymic *i*NKT cells and affected thymic *i*NKT1, *i*NKT2, and *i*NKT17 differentiation (Burocchi et al., 2015; Wang et al., 2021). However, loss of miR-155 didn’t affect development of *i*NKT cells (Frias et al., 2018; Wang et al., 2021).

PLZF protein regulates MAIT cell maturation (Pellicci et al., 2020). In PLZF-null mice, stage 3 MAIT cells are completely absent in the thymus and peripheral organs (Rahimpour et al., 2015; Koay et al., 2016), and the PLZF-encoding gene *Zbtb16* is required for cutaneous MAIT cell development (Constantinides et al., 2019). Approximately 70% thymic MAIT cells express PLZF, which is consistent with the overall frequency of stage 3 MAIT cells, implying PLZF^+^ cells represent MAIT cells with functional maturation (Winter et al., 2019). Thus, unlike *i*NKT cells that already express substantial amounts of PLZF at the earliest stage of development (Savage et al., 2008), MAIT cells start to express PLZF at functional maturation stage. Our recent study indicated that miR-155 up-regulation promotes PLZF expression during *i*NKT cell development (Wang et al., 2021). In contrast to *i*NKT cells, our current study showed that overexpression of miR-155 downregulated PLZF expression in thymic MAIT cells, suggesting a differential role in PLZF expression by miR-155 in *i*NKT cells and MAIT cells. However, miR-155 overexpression doesn’t affect PLZF expression in the peripheral MAIT cells. How miR-155 regulates thymic PLZF expression and MAIT cell development needs to be further investigated in the future.

Few genes have been reported to regulate the differentiation of MAIT cells. CCR7 is selectively required for the differentiation of MAIT17 subset (Koay et al., 2019b), and miR-181a/b-1 downregulates expression of RORγt and T-bet only in the thymus, but is dispensable for peripheral differentiation (Winter et al., 2019). Here we found that overexpression of miR-155 interrupted differentiation of MAIT cells by blocking MAIT17 and promoting MAIT1 cell, which is in paralleled with our previous result of miR-155 on *i*NKT cells. Overexpression of miR-155 dramatically reduces *Rictor* expression (an obligatory component of mTORC2; Laplante and Sabatini, 2009) and up-regulation of miR-155 diminishes *i*NKT17 cells in thymus and lymph nodes through targeting *Rictor* (Wang et al., 2021). Thus, we suspect that, in the process of MAIT cell differentiation, overexpression of miR-155 may repress the expression of *Rictor* and block MAIT17 differentiation by the same target signaling pathway identified in *i*NKT cells.

Interestingly, MAIT 17 blockage and MAIT1 promotion in thymus were also found from miR-155 deficiency mice. So, we proposed a hypothesis that normal expression of miR-155 is essential to maintain the normal functional differentiation of MAIT cells. Either overexpression or deletion of miR-155 can modify MAIT cell differentiation by targeting different genes. Up-regulation of miR-155 may inhibit the genes (such as *Rictor*) involved in MAIT17 cells to block MAIT17 cells differentiation, while deletion of miR-155 may increase the genes expression involved in MAIT1 cells to promote MAIT1 cells differentiation. Thus, adequate level of miR-155 is very important to the balance of MAIT1 and MAIT 17 differentiation.

Unlike *i*NKT cells that almost all cells are CD8 negative, four MAIT cell subsets can be identified based on expression of CD4 and CD8. CD4^+^CD8^–^, CD4^–^CD8^+^, CD4^–^CD8^–^, and CD4^+^CD8^+^ in the thymus, and 3 major subsets CD4^+^CD8^–^, CD4^–^CD8^+^, CD4^–^CD8^–^ MAIT cells in peripheral tissues. CD4^–^CD8^–^ MAIT cell is the majority subset in all the tissues, but CD4^+^CD8^–^ or CD4^–^CD8^+^ subsets also account for some proportion in the thymus, spleen, LNs, and liver (Rahimpour et al., 2015; Constantinides et al., 2019; Winter et al., 2019). Here we examined the different subsets of MAIT cells based on expression of CD4 and CD8 on MAIT17 and MAIT1 cells, and found that CD4^–^CD8^–^ cell subset was abundant in MAIT17 cells and the CD4^–^CD8^+^ cell subset was enriched in MAIT1 cells in the thymus, spleen, LNs, liver, and lung in WT mice. Overexpression of miR-155 decreased CD4^–^CD8^–^ subset and increased CD4^–^CD8^+^ subset both in MAIT1 and MAIT17 cells from thymus and peripheral organs, indicating that miR-155 may regulate MAIT cell sub-lineage differentiation and CD4^–^CD8^–^/CD4^–^CD8^+^ subsets.

We do realize there are some limitations in our current study, including the dissection of miR-155 expression pattern during thymic development (from stage 1 to stage 3) and its targeted signaling pathways involved in MAIT cell development and function. However, due to very limited MAIT cells we can get, so it is very difficult to get enough thymic MAIT cells from miR-155 KI mice and miR-155KO mice to perform RNA-seq or miRNA-seq analysis. Furthermore, given that miRNAs repress their target genes post-transcriptionally and few protein coding genes have been identified to regulate MAIT cell development and function so far, it makes defining miR-155 targeting genes in MAIT cells even harder. Finally, the cytokines production capability (PMA/Ionomycin stimulation) detected in our study only reflects downstream of TCR signaling pathway. TCR-dependent MAIT cell activation, including 5-OP-RU-mediated activation, need to be investigated in future. In conclusion, our results indicate that adequate miR-155 expression is required for normal MAIT1 and MAIT17 cell development and function.

## Data Availability Statement

The original contributions presented in the study are included in the article/supplementary material, further inquiries can be directed to the corresponding author/s.

## Ethics Statement

The animal study was reviewed and approved by the Institutional Animal Care and Use Committee.

## Author Contributions

Q-SM and LZ conceived and designed the experiments. TL, KS, and JW performed the experiments. Q-SM, LZ, and TL analyzed the data. QY did genotyping. TL, LZ, and Q-SM drafted the manuscript, which was commented on by all authors.

## Conflict of Interest

The authors declare that the research was conducted in the absence of any commercial or financial relationships that could be construed as a potential conflict of interest.
